# LSD 4.0: an improved database for comparative studies of leaf senescence

**DOI:** 10.1186/s43897-022-00045-w

**Published:** 2022-10-10

**Authors:** Jie Cao, Yang Zhang, Shuya Tan, Qi Yang, Hou-Ling Wang, Xinli Xia, Jingchu Luo, Hongwei Guo, Zhang Zhang, Zhonghai Li

**Affiliations:** 1 National Engineering Research Center for Tree Breeding and Ecological Restoration, College of Biological Sciences and Technology, Beijing Forestry University, Beijing, 100083 China; 2 National Genomics Data Center, Beijing, 100101 China; 3 China National Center for Bioinformation, Chinese Academy of Sciences, Beijing, 100101 China; 4 University of Chinese Academy of Sciences, Beijing, 100049 China; 5 College of Life Sciences, Peking University, Beijing, 100871 China; 6 Center for Bioinformatics, Peking University, Beijing, 100871 China; 7 Department of Biology, Institute of Plant and Food Science, Southern University of Science and Technology (SUSTech), Shenzhen, 518055 Guangdong China

Leaf senescence is the final stage of leaf development and involves the active degradation and dynamic transfer of its cellular components to newly growing and storage tissues, which contributes to plant fitness (Gan and Amasino, 1997; Lim et al., 2007). The genetic modification of leaf senescence has emerged as a promising strategy for improving nutritional traits and stress tolerance in plants (Rivero et al., 2007). Efforts to dissect the molecular mechanisms underpinning leaf senescence reveal that it is a highly coordinated process regulated by a large number of senescence-associated genes (SAGs) (Guo and Gan, 2005; Lim et al., 2007). Functional studies of these SAG genes through reverse genetics strategies and identification of senescence-altered mutants through forward genetic screening have deepened the understanding of leaf senescence (Guo et al., 2021). To facilitate systematic and comparative studies of leaf senescence, we developed the leaf senescence database (LSD) in 2010, and updated it in 2014 and 2019 (Li et al., 2020), respectively. This database had been widely used for systematic identification and functional studies of SAGs in agronomically important crops.

In the past 3 years, many advances had been made in leaf senescence research. As a result, thousands of SAGs have been identified in new plant species and hundreds of new genes have been found to be as functional SAGs. To cover these advances and extend the functionality of the previous LSD, we updated our database to the new version LSD 4.0 (LSD, https://ngdc.cncb.ac.cn/lsd/). We have improved the original database and added some new features. The updated database provides useful resources for revealing the regulatory mechanisms of leaf senescence through comparative biological strategies and for improving the quality and yield of crop plants by fine-tuning leaf senescence process.

The updated database contains 31,214 genes and 1,037 mutants from 86 species, an extension from the previous version containing 5,853 genes and 617 mutants from 68 species, increased by 5.3-fold and 1.7-fold, respectively (Fig. 1A-C). We performed manual curation to retrieve a wide range of information, including gene name, locus name, GenBank ID, PubMed ID, mutant, species, senescence-associated phenotype, the effect on leaf senescence and evidence. We made extensive annotations for these SAGs through computational approaches, including Gene Ontology, DNA and protein sequences, protein–protein interactions, miRNA interaction information, as well as ortholog groups.

**Fig. 1:**
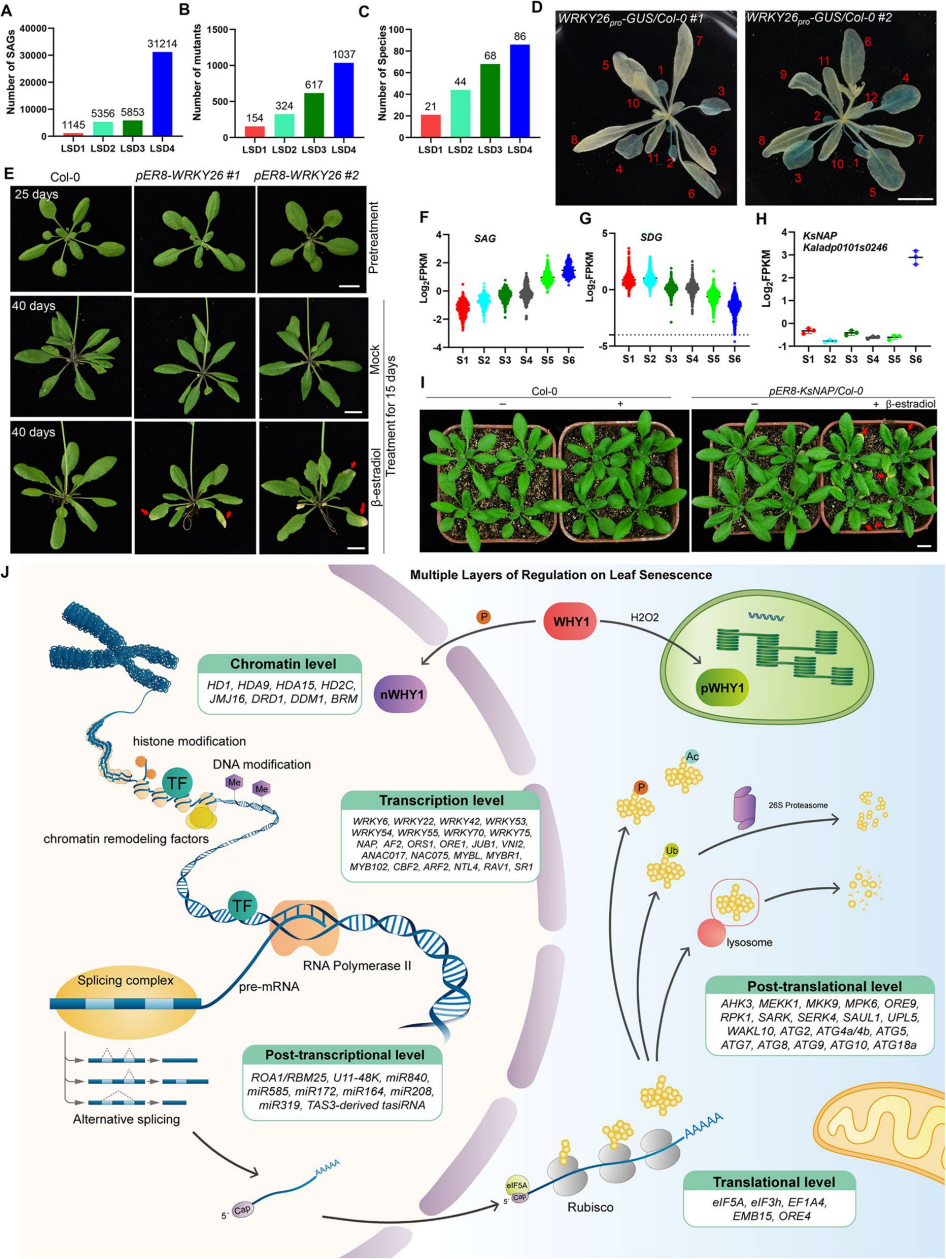
Comparison of entries changes in four versions of LSD and functional assessment of SAG. **A-C** Histogram illuminates the comparisons of gene number (**A**), mutant number (**B**) and species number (**C**) among four versions of the database. **D-E** WRKY26 is a positive regulator of leaf senescence. Histochemical analysis of the rosette leaves of 28-d-old transgenic plants of *WRKY26*_*pro*_*-GUS/Col-0* (#1 and #2). The numbers were labeled according to the order of appearance of the rosette leaves. The larger the number, the younger the leaf. Bar, 1 cm (**D**). Inducible overexpression of *WRKY26* leads to premature leaf senescence in 40-day-old *pER8-WRKY26/Col-0* transgenic plants (#1 and #2) after spraying with 50 μM β-estradiol for 15 days. Red arrows indicate leaves that have turned yellow and senescent. Bar, 1 cm (**E**). **F-I** Identification and functional analysis of SAGs in *Kalanchoe serrata*. Identification of 987 SAGs (**F**) and 857 SDGs (**G**) in *Kalanchoe serrata* Plants. S1-S6, six different developmental stages. Expression level of *KsNA*P at different stage (**H**). Inducible overexpression of *KsNAP* promotes leaf senescence in 28-day-old Arabidopsis plants after spraying with 50 μM β-estradiol. Red arrows indicate leaves that have turned yellow and senescent. Bar, 1 cm (**I**). **J** The multi-layered controls of leaf senescence. The onset and progression of leaf senescence is finely controlled by multiple layers of regulation, mainly including chromatin-mediated, transcriptional, post-transcriptional, translational and post-translational levels

So far, the function of the vast majority of SAG genes is unknown, which may explain why the mechanism of leaf senescence remains unclear. Given the critical role of transcription factors in the regulation of leaf senescence, we proposed to construct dominant or dominant-negative plants by overexpressing senescence-associated transcription factors with unknown function. For example, we found that WRKY26 is a functional SAG that positively regulates leaf senescence (Fig. 1D and E). To investigate the correlation between *WRKY26* expression and leaf senescence, we constructed transgenic plants expressing the GUS gene driven by its promoter (*WRKY26*_*pro*_*-GUS/Col-0*) using the primers listed in Table S1. GUS staining analysis revealed that *WRKY26* showed higher expression in older leaves (Fig. 1D). Next, we generated inducible overexpression plants *pER8-WRKY26* to assess its regulatory role in leaf senescence (Fig. S1A). Some leaves of transgenic plants started to turn yellow after induction of *WRKY26* gene expression by application of β-estradiol compared with control plants with lower levels of chlorophyll content and photochemical efficiency of PSII (Fv/Fm) (Fig. 1E; Fig. S1B and S1C), suggesting that WRKY26 is a positive regulator of leaf senescence. Further research is needed to reveal the underlying regulatory mechanism.

Currently, plants used for leaf senescence research include C3 plants such as rice and wheat, and C4 plants such as sorghum and maize, but no data is available in Crassulacean acid metabolism (CAM) plants. C3, C4 and CAM are three different processes that plants use to fix carbon during the process of photosynthesis. CAM is a specialized mode of photosynthesis that exploits a temporal CO_2_ pump with nocturnal CO_2_ uptake and concentration to reduce photo-respiration, improve water-use efficiency, and optimize the adaptability of plants to hot and dry areas (Borland et al., 2009). To provide the transcriptomic picture of leaf senescence in CAM plants, we selected *Kalanchoe serrata* in the genus Kalanchoë mainly because of their great variation in leaf morphology at different leaf age stages (Garces et al., 2007) (Fig. S2). In total, 987 SAGs and 857 senescence down-regulated genes (SDGs) were identified according to their increased or decreased expression levels as leaves age, respectively (Fig. 1F and G). Inducible overexpression of a senescence-associated NAC transcription factor *KsNAP* (Kaladp0101s0246) (Fig. 1H; Fig. S3A), an ortholog of AtNAP/NAC029 (Guo and Gan, 2006), accelerated Arabidopsis leaf senescence process demonstrated by the earlier leaf yellowing, lower levels of chlorophyll content and Fv/Fm in β-estradiol-treated *pER8-KsNAP* plants (Fig. 1I; Fig. S3B and S3C), indicative of the functional conservation of NAP in the regulation of leaf senescence across plant species. Transcriptomic data in *Kalanchoe serrata* were integrated to the updated database to provide comprehensive gene expression profiles for leaf senescence research in CAM plants.

Additionally, to help researchers discover new regulators of leaf senescence, the information on senescence-associated proteins (SAP), with enhanced protein levels as leaves age, was integrated into the updated database. Transcription factors play important roles in the regulation of leaf senescence, but their target genes are mostly unknown. Therefore, ChIP-Seq or DAP-seq data of senescence-associated transcription factors were added into the database to help researchers reveal their regulatory mechanisms. Multi-omics studies have revealed that leaf senescence is subjected to multiple layers of regulation (Woo et al., 2013) (Fig. 1J). Recent studies reported that alternative splicing, a type of post-transcriptional regulation of gene expression, is involved in regulating leaf senescence (Wang et al., 2021). Thus, senescence-associated alternative splicing variants (Sen-ASVs) were added in LSD 4.0. These newly added data provide important clues for researchers to elucidate the molecular regulatory mechanisms of leaf senescence.

LSD 4.0 is the only available resource specialized in leaf senescence, providing a convenient way to study the multiple-layer regulation and evolution of leaf senescence through comparative biological strategies. To better serve the leaf senescence research community, we will continue to improve the database from the following aspects. (i) To integrate newly identified *SAG*s and mutant information via manual curation. (ii) To investigate the function of *SAG*s by reverse genetics approach and integrate their phenotype information into LSD. (iii) To identify *SAG*s at cellular level by using single-cell transcriptome sequencing (scRNA-seq) technology, which will deepen our understanding of leaf senescence. (iv) To identify *SAG*s in an aquatic plant *Wolffia Australiana* using multi-omics techniques and integrate into LSD, which will provide resource data for comparative studies of leaf senescence in terrestrial and aquatic plants. (v) To update and improve web interfaces according to the suggestions from users. Taken together, the emergence of new research techniques will lead to an increasing amount of data related to leaf senescence, and we then continue to upgrade this database to better serve the research community.

### Supplementary Information

**Additional file 1. **

## Data Availability

The data and materials will be available upon reasonable request.
