# Supplementary material for: LSD 4.0: an improved database for comparative studies of leaf senescence
Source: Mol Hortic. 2022 Oct 10;2:24. doi: 10.1186/s43897-022-00045-w (PMC10515038; doi:10.1186/s43897-022-00045-w)
Supplement: Supplementary file 1 — Additional file 1. [file 43897_2022_45_MOESM1_ESM.doc]

**Supplementary Information**

**Materials and methods**

**Plant materials and growth conditions**

*Arabidopsis thaliana* Columbia-0 (Col-0) and *Kalanchoe serrata* were used in this study. Plants were grown at 22 °C under long day conditions (16 h light/8 h dark).

**Plasmid construction and transformation**

To generate the estradiol-induced *KsNAP* or *WRKY26* overexpression lines, the CDS of *KsNAP* or *WRKY26* was amplified and then introduced into the pER8 vector (Zuo et al., 2000), respectively. To generate *WRKY26_pro_-GUS/Col-0*, the promoter region of WRKY26 was obtained and inserted into the pBI101 vector (Jefferson et al., 1987) as described previously. The floral dip method was used to construct transgenic plants (Clough and Bent, 1998).

**RNA extraction and quantitative Real-Time PCR analysis**

Total RNA extraction and quantitative RT-PCR (qPCR) analysis were performed as previously described (Wang et al., 2022), and the relative mRNA quantities were calculated using the ΔΔCT method (Schmittgen and Livak, 2008).

**RNA-sequencing analysis**

The leaves of *Kalanchoe serrata* plants were collected and ground into a powder in liquid nitrogen. Total RNA was extracted as previously described (Zhang et al., 2022), and RNA-Seq data were generated with an Illumina HiSeq™ 2500 sequencing platform (Biomarker Ltd., Beijing, China). The reads were then mapped to the *Kalanchoë fedtschenkoi* reference genome using the Hisat2 algorithm. The differentially expressed genes (|Log2 (fold change)| > 1.0, P < 0.05) were deposited in the website of Leaf Senescence Database (https://ngdc.cncb.ac.cn/lsd/).

**Histochemical analysis and quantification of GUS activity**

GUS staining was carried out as described previously (Jefferson et al., 1987). 4-methylumbelliferyl-β-D-glucuronide (4-MUG; Sigma-Aldrich) was used the substrate to quantify the activity of GUS.

**Measurement of chlorophyll contents and Fv/Fm**

Chlorophyll meter Konica Minolta SPAD502 Plus (Sakura-machi, Hino-shi Tokyo, Japan) was used to measure chlorophyll contents, and MultiSpeQ instrument (East Lansing, MI, United States) was used to measure Fv/Fm according to instructions.

**Abbreviations**

LSD: leaf senescence database; SAG: senescence-associated gene; CLE: CLAVATA3/ESR-RELATED; ROS: reactive oxygen species; ABA: abscisic acid; ABI5: ABA INSENSITIVE5; NYC1: NON-YELLOW COLORING1; IR: intron retention; GI: GIGANTEA; Sen-ASVs: senescence-associated alternative splicing variants; SAP: senescence-associated proteins; TF: Transcription factors; Sen-TFs: senescence-associated TFs; CAM: Crassulacean acid metabolism; SDGs: senescence down-regulated genes; Fv/Fm: photochemical efficiency of PSII; scRNA-seq: single-cell transcriptome sequencing

**References**

**Clough SJ, Bent AF** (1998) Floral dip: a simplified method for Agrobacterium-mediated transformation of Arabidopsis thaliana. Plant J **16:** 735-743

**Jefferson RA, Kavanagh TA, Bevan MW** (1987) GUS fusions: beta-glucuronidase as a sensitive and versatile gene fusion marker in higher plants. EMBO J **6:** 3901-3907

**Schmittgen TD, Livak KJ** (2008) Analyzing real-time PCR data by the comparative C(T) method. Nat Protoc **3:** 1101-1108

**Wang HL, Yang Q, Tan S, Wang T, Zhang Y, Yang Y, Yin W, Xia X, Guo H, Li Z** (2022) Regulation of cytokinin biosynthesis using PtRD26pro -IPT module improves drought tolerance through PtARR10-PtYUC4/5-mediated reactive oxygen species removal in Populus. J Integr Plant Biol **64:** 771-786

**Zhang Y, Tan S, Gao Y, Kan C, Wang HL, Yang Q, Xia X, Ishida T, Sawa S, Guo H, Li Z** (2022) CLE42 delays leaf senescence by antagonizing ethylene pathway in Arabidopsis. New Phytol **235:** 550-562

**Zuo J, Niu QW, Chua NH** (2000) Technical advance: An estrogen receptor-based transactivator XVE mediates highly inducible gene expression in transgenic plants. Plant J **24:** 265-273

**Supplementary Figures**

**
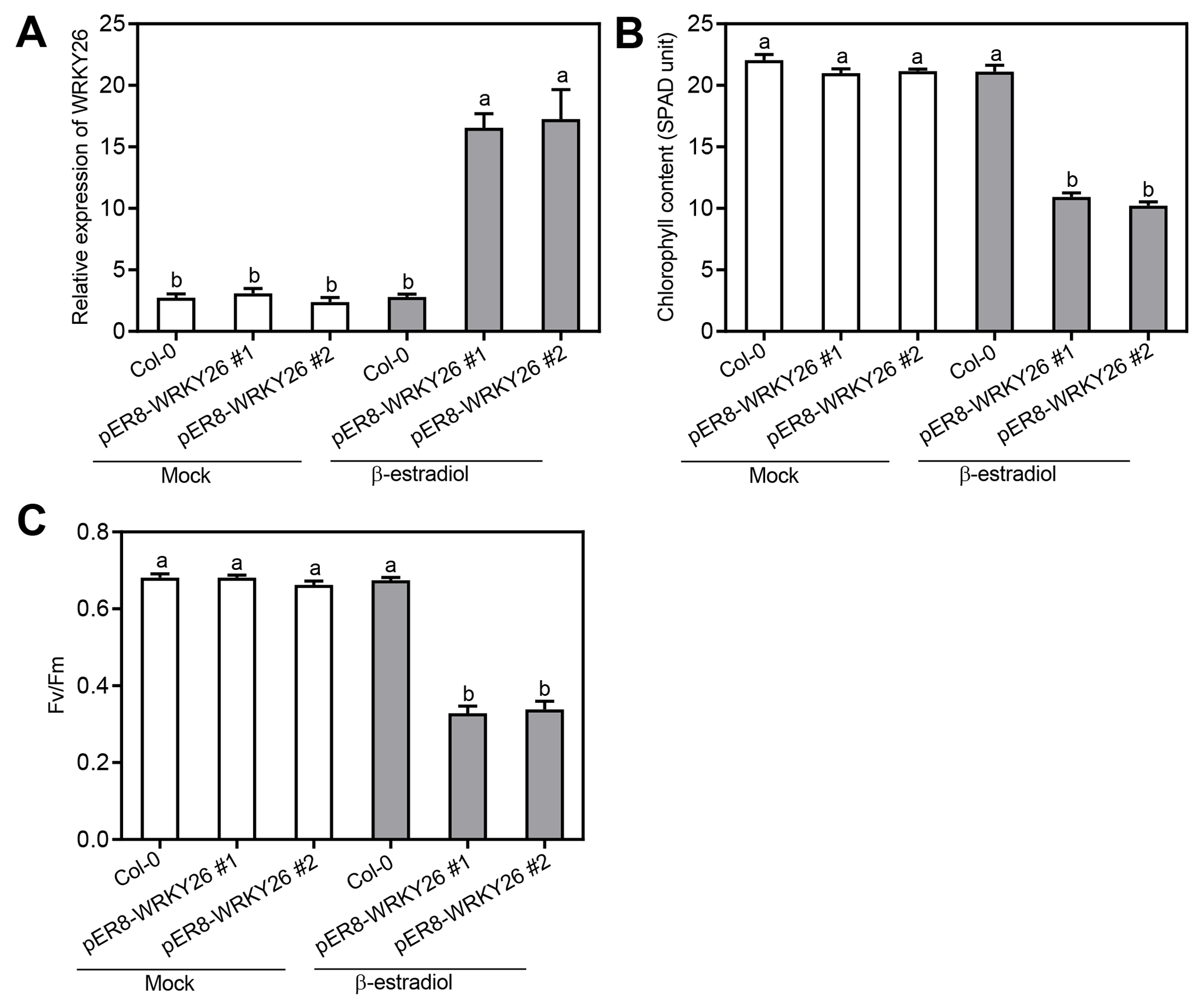
**

**Fig. S1** Inducible overexpression of *WRKY26* accelerates leaf senescence. **A** RT-qPCR analysis of *WRKY26* expression in the leaves of 14-day-old Col-0 and *pER8-WRKY26/Col-0* #1 and #2 plants after spraying with water (Mock) or 50 µM β-estradiol. **B-C** Measurement of chlorophyll content (**B**) and photochemical efficiency of PSII (Fv/Fm) (**C**) in the fourth leaves of 40-day-old plants of Col-0, *pER8-WRKY26* #1 and #2 after spraying with water (Mock) or 50 µM β-estradiol.

**
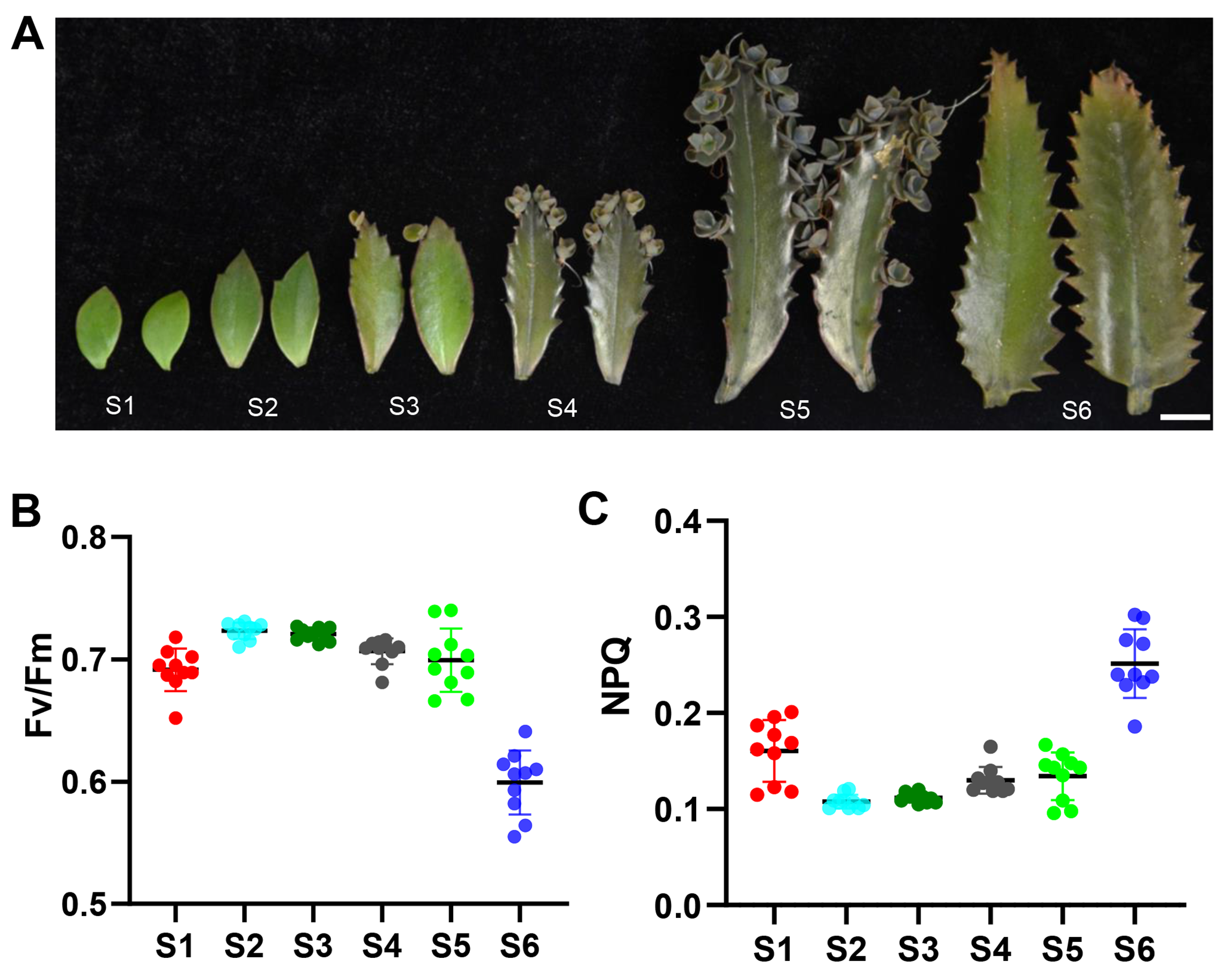
**

**Fig. S2** Leaves of *Kalanchoe serrata* plants used for transcriptome analysis. **A** Representative leaf of six different developmental stages in *Kalanchoe serrata* plants. The leaves were used for RNA extraction after the plant-lets were removed. Bar, 1 cm. **B-C** Measurement of photochemical efficiency of PSII (Fv/Fm) (**B**) and non-photochemical quenching (NPQ) (**C**) in the leaves of *Kalanchoe serrata* Plants at six different developmental stages (S1-S6).


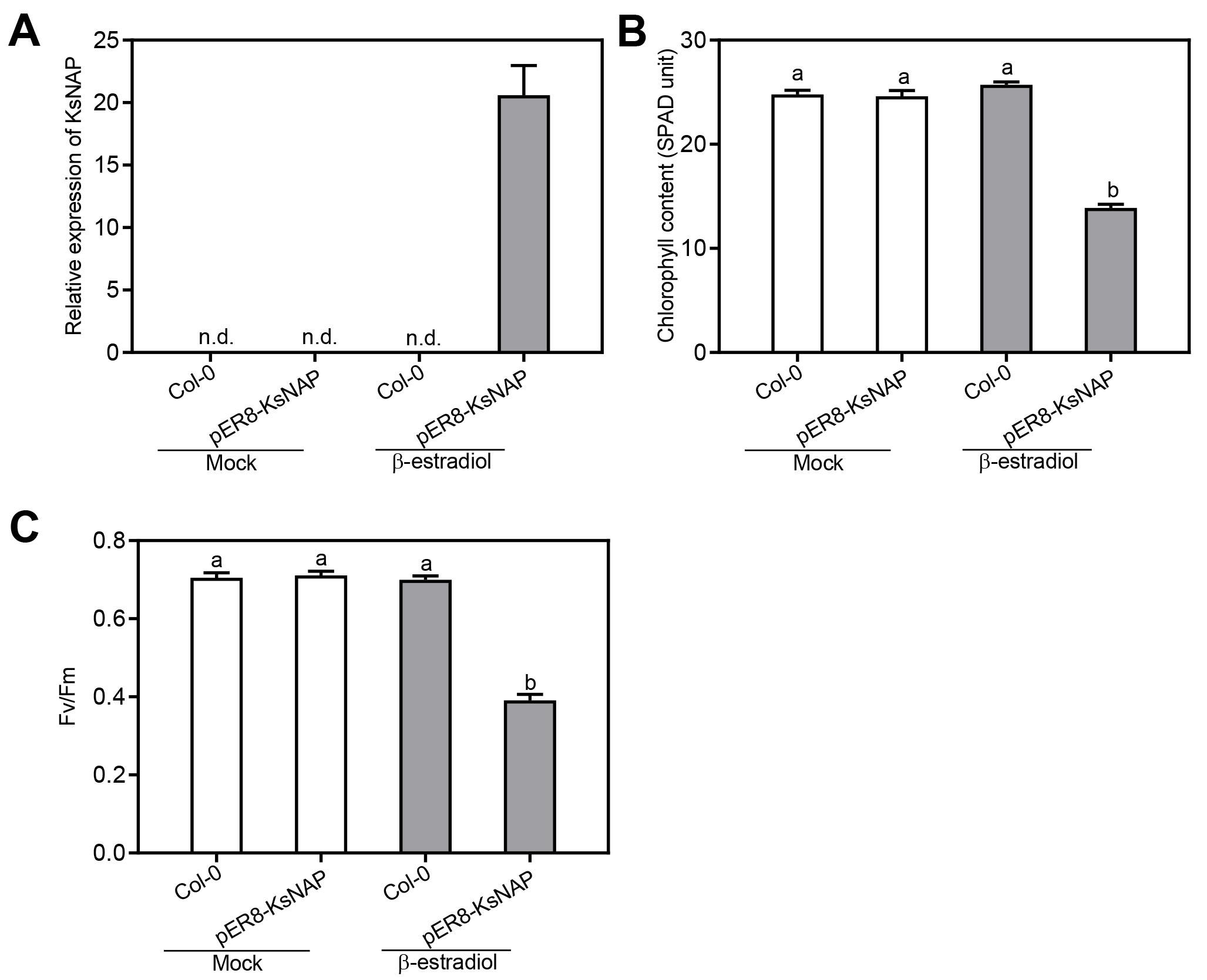


**Fig. S3** Inducible overexpression of *KsNAP* promotes leaf senescence in Arabidopsis. **A** RT-qPCR analysis of *KsNAP* expression in the leaves of 14-day-old Col-0 and *pER8-KsNAP/Col-0* plants after spraying with water (Mock) or 50 µM β-estradiol. n.d., not detected. **B-C** Measurement of chlorophyll content (**B**) and photochemical efficiency of PSII (Fv/Fm) (**C**) in the fourth leaves of 28-day-old Col-0 and *pER8-KsNAP/Col-0* plants after spraying with water (Mock) or 50 µM β-estradiol.

**Table S1. Primers used in the study.**

| **Names** | **Sequence (5'-3')** |
| --- | --- |
| **Plasmid construction** | |
| WRKY26-F | ATACGCGTTAATTAACTAGTATGGGCTCTTTTGATCGCCA |
| WRKY26-R | GGAGGCCTGGATCGACTAGTTTATGTCTCTGTTTTTCCAA |
| WRKY26-GUS-F | GAAAGTCCAACTGCTTTGAA |
| WRKY26-GUS-R | GGAAAGGATGTTTCCAAGGA |
| KsNAP-F | ATACGCGTTAATTAACTAGTATGAAGATGGGATCAGGACA |
| KsNAP-R | GGAGGCCTGGATCGACTAGTTCATTGGAACTCGTACAGTG |
| **Gene expression analysis** | |
| qWRKY26-F | ATGGGCTCTTTTGATCGCCA |
| qWRKY26-R | GCAAACCGTTATTGTTATAG |
| qKsNAP-F | ATGAAGATGGGATCAGGACA |
| qKsNAP-R | GTTCTCCCCGAACTCCGC |
| qUBC21F | TCAAATGGACCGCTCTTATC |
| qUBC21R | CACAGACTGAAGCGTCCAAG |
